# Effect of Nutrition Education Based on Health Belief Model on Nutritional Knowledge and Dietary Practice of Pregnant Women in Dessie Town, Northeast Ethiopia: A Cluster Randomized Control Trial

**DOI:** 10.1155/2018/6731815

**Published:** 2018-06-21

**Authors:** Tona Zema Diddana, Gezahegn Nigusse Kelkay, Amanuel Nana Dola, Abinet Arega Sadore

**Affiliations:** ^1^School of Nutrition, Food Science and Technology, Hawassa University, Hawassa, Ethiopia; ^2^Faculty of Chemical and Food Engineering, Bahir Dar Institute of Technology, Bahir Dar, Ethiopia; ^3^School of Public Health, Jimma University, Jimma, Ethiopia

## Abstract

**Background:**

In Ethiopia, poor dietary practice among pregnant women ranges from 39.3 to 66.1%. Limited nutritional knowledge and wrong perception towards dietary behaviours were underlying factors. Hence, this study was aimed to determine the effect of nutrition education based on Health Belief Model on nutritional knowledge and dietary practice of pregnant women in Dissie town, northeast Ethiopia, 2017 GC.

**Methods:**

Community-based cluster randomized control trial was employed. A total of 138 pregnant women participated. Nutrition education was given using Health Belief Model (HBM) theory and general nutrition education for intervention and control group, respectively. The baseline and endline nutrition knowledge and dietary practice was assessed using knowledge and dietary practice questions. HBM construct was assessed using five-point likert scale. Data were analyzed using SPSS version 20. Student's *t*-tests and chi-square tests were used. At 95% confidence level, *P* < 0.05 was considered statistically significant.

**Result:**

The mean pre- and postintervention nutritional knowledge was 6.9 and 13.4, and good dietary practice was 56.5% and 84.1% in intervention group, respectively. The increase in mean nutritional knowledge was statistically significant (*P* < 0.001). In control group, the pre- and postintervention mean nutritional knowledge was 7.4 and 9.8, and good dietary practice was 60.9% and 72.5%, respectively. There was significant difference (*P* < 0.05) in mean nutritional knowledge and proportion of good dietary practices between two groups at endline, but the difference was not significant (*P* > 0.05) at baseline. There was significant (*P* < 0.001) improvement in the scores of HBM constructs in intervention group.

**Conclusion and Recommendations:**

Providing nutrition education based on Health Belief Model improves nutritional knowledge and dietary practices of pregnant women. Hence, governmental, nongovernmental organization, health extension workers, and other health-care provider should include Health Belief Model construct into existing nutrition education programs. Moreover, government should incorporate HBM theory into national nutrition education guidelines.

## 1. Introduction

Pregnancy is the most crucial nutritionally demanding period of every woman's life. Appropriate nutrient intake during this period has a critical role in fetal development [1] and better maternal nutritional status [2]. Failure to receive necessary micro- and macronutrient during this period will result in undernutrition and adverse pregnancy outcome [3]. Undernutrition during pregnancy wields both short- and long-term effects on the health of an infant by programming the infant's development [4], increases the risk of noncommunicable diseases [5], and is intimately related to the survival of both mothers and their babies [6]. Undernourished mothers are more vulnerable to diseases, encounter more miscarriages, and give birth to underweight children whose survival is at risk [6, 7].

Ethiopia is one of the countries with high burden of maternal and child undernutrition. Available literatures in Ethiopia indicated that poor dietary practice among pregnant women in Ethiopia ranged from 39.3 to 66.1% [8–10], and undernutrition was 15.2–35.5% [9–11]. This might be attributable to poor knowledge and wrong perception towards dietary behaviours. In 2016, 22.4% of women were undernourished in Ethiopia and 22.9% in Amhara region, the region where this study was conducted. About 23.6% of women aged 15–49 are anemic, with 17.8% having mild anemia, 5% having moderate anemia, and 0.8% having severe anemia. The prevalence of anemia in Amhara region was 17.2% with 14.6% mild anemia, 2.4% with moderate anemia, and 0.1% with severe anemia [12].

Many women in developing countries restrict food intake during pregnancy for different reasons such as to have smaller infants on the premise that smaller infants will carry a lower risk of delivery complications [13] due to cultural influence and premise that it makes fetus big and challenge during delivery [8, 14]. Restriction of food intake and inappropriate nutrition practices in combination with environmental and socioeconomic factor and infections is common causes of maternal mortality, low birth weight, and intergrowth retardation [15, 16]. For instance, iodine deficiency causes still birth, cretinism, and mental retardation [17, 18], iron deficiency causes anemia and increases the risk of mortality [19], vitamin A deficiency causes night blindness, intrauterine growth retardation, and low birth weight [20, 21]. Furthermore, low weight gain during pregnancy results in delivery of infants too small for gestational age leading to neonatal mortality and morbidity [22], failure in growth, slow cognitive development, and chronic diseases in adulthood [23].

The world health assembly targeted to reduce under five stunting, anemia in women of reproductive age, and low birth weight by the rate of 40%, 29.4%, and 30% by 2030, respectively [24]. Without improving nutritional knowledge, dietary practices, and perceived dietary behavior during pregnancy, it may be challenging to achieve this target since optimal physical growth and cognitive development are founded on maternal and child nutrition in the first 1000 days.

**Figure 1:**
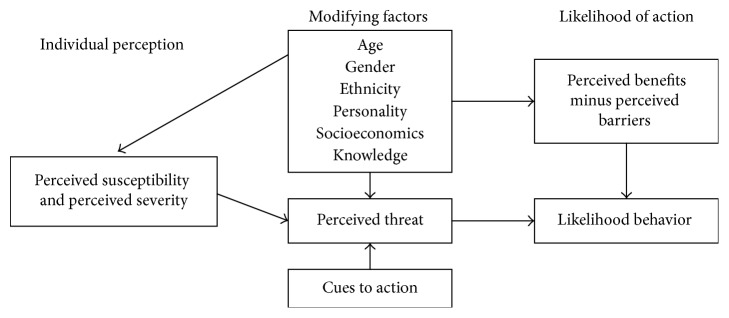
Health Belief Model theory components and linkages.

The Health Belief Model (HBM) is the most commonly used theory in health education and health promotion to explain change and maintenance of health-related behaviors and as a guiding framework for health behavior interventions [25]. It is a psychological model that attempts to explain and predict health behaviors through focusing on the attitudes and beliefs of individuals. The underlying concept of the HBM is that health behavior is determined by personal beliefs or perceptions about a disease/health condition and the strategies available to decrease its occurrence. It contains several primary constructs/concepts perceived susceptibility, severity, benefit, barrier, and self-efficacy that predict why people will take action to prevent, to screen for, or to control illness conditions (Figure 1) [26].

By using learning methods to counteract and reduce existing barriers, they are able to mitigate these adverse effects. Moreover, people can change their attitudes, and the range of positive behaviors will increase. With this aim, this study was conducted to determine the effect of nutrition education based on Health Belief Model on nutritional knowledge and dietary practice of pregnant women in Dissie town, northeastern Ethiopia, 2017 GC.

## 2. Materials and Methods

### 2.1. Description of Study Area

This study was conducted in Dissie town, northeast Ethiopia. The town is 401 km away from Addis Ababa, capital of Ethiopia. According to the Dessie town administration office report of 2017 GC, the town has total population of 216,384, of which 46.86% were male and 53.14% were female. There were 10 subcities and 10 health-care institutions that provide health-care services in the town. Geographically, the town is located at 11°8′N latitude and 39°38′E longitude with an elevation between 2470 m and 2550 m above the sea level. The town mainly characterized by woina-dega climatic condition with annual average temperature 15.2°C and 1145 mm rainfall of the town.

### 2.2. Study Design and Period

A community-based cluster randomized control trial was employed from May to September 2017 GC.

### 2.3. Source Population

The source population was all pregnant women who were permanent residents in Dissie town during the study period.

### 2.4. Study Population

The study population was pregnant women who were permanent residents in four selected subcities (clusters) of Dissie town during the study period.

### 2.5. Inclusion Criteria

All pregnant women who were permanent residents in the town and apparently healthy (self-reported) at the time of baseline data collection were included.

### 2.6. Exclusion Criteria

Those pregnant women who were above four months of pregnancy at the baseline data collection were excluded.

### 2.7. Sample Size Determination

The sample size (*n*) was determined based on the difference between two proportions of dietary practice. The prevalence of good dietary practice (*P*_2_ = 40.1%) was used from previous study [16], for control group. By hypothesizing that the prevalence of good dietary practice would be improved by 25% in this study, proportion in intervention group (*P*_1_) was 65.1% (25% + 40.1%). The level of confidence (*α*) was taken to be 0.05 (*Z*_*α*/2_ = 1.96); the power (1−*β*) 100% was taken to be 80% (*Z*_2_ = 0.84); and 10% contingency for loss to follow-up was added. Design effect for effect of cluster randomization was ignored because when using design effect, the sample size became higher than study population. Intracluster correlation coefficient (ICC) was calculated to quantify the degree of similarity in the responses of individuals from the same cluster for the primary outcome variables. This gave ICC of 0.132 and −0.024 for dietary practices and nutritional knowledge, respectively. This suggested that clustering was safe to ignore for studied population. Hence, using the following formula, the total final sample size included in this study was 138 (69 for each group) pregnant women:

(1) $$\begin{array}{c} n=\frac{{\left(Z_{1}+Z_{2}\right)}^{2}\;*\;2P\left(1-P\right)}{{\left(P_{2}-P_{1}\right)}^{2}}. \end{array}$$

### 2.8. Sampling Technique and Procedure

**Figure 2:**
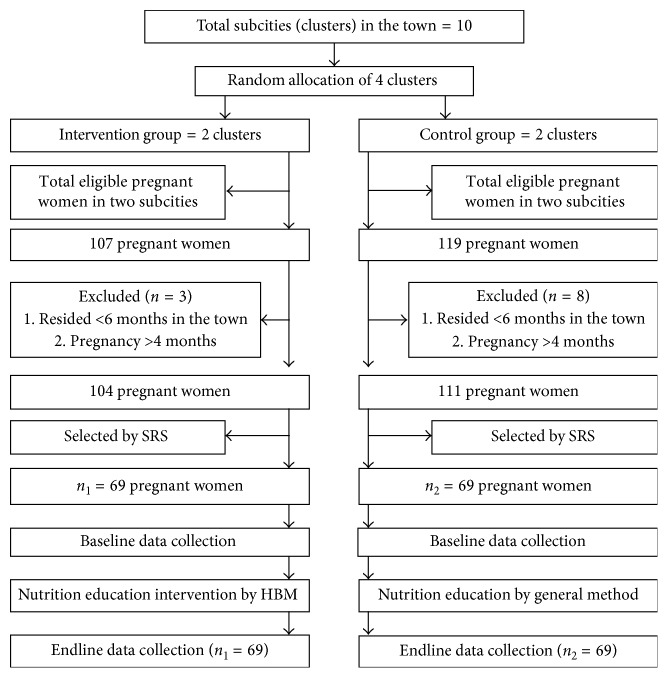
Sampling procedure of pregnant women. SRS = simple random sampling; *n*_1_ = sample size for intervention group; *n*_2_ = sample size for control group; HBM = Health Belief Model.

The sampling procedure is indicated in Figure 2. Pregnant women living in four randomly selected subcities (clusters) were identified from health extension workers' records. Those not registered in health extension records were identified through house to house visit. Sampling frame was created, and participants were selected by simple random sampling method. Random numbers were generated using ENA-for-SMART software.

### 2.9. Data Collection Instruments and Procedures

Socioeconomic and demographic information were collected only at baseline. Stage of pregnancy, Health Belief Model constructs, nutrition knowledge, and dietary practices were collected both at baseline and end of nutrition education. Data were collected using structured questionnaires. Nutritional knowledge was collected by using 15 nutrition knowledge questions. Participants were given score = 1, if they correctly answer knowledge question, and score = 0, if they did not correctly answer the question. Similarly, dietary practices data were collected by using 17 dietary habit questions. Participants were given score = 1, if they correctly answer question, favorable or healthy for dietary practice, and score = 0, if they did not correctly answer the question, not favorable or healthy for dietary practice. Items of Health Belief Model constructs were measured using five-point likert scale (strongly agree = 5; through strongly disagree = 1). Then, the value of each likert scale scored by participants for each question was summed and the average was calculated.

### 2.10. Data Quality Control

The questionnaire was first prepared in English, and then translated to local language, Amharic (national language of Ethiopia). Two days of training was given to data collectors and supervisors. Pretesting was done on 5% of calculated sample size in the area other than study clusters. The data were monitored daily during collection. Collected questionnaires were checked for completeness and consistency during interview and at the end of each day.

### 2.11. Nutrition Education Intervention

Nutrition education intervention was given to pregnant women between 1 and 4 months at baseline. The education was given every 15 days for 5 consecutive months. For intervention group, education intervention was given based on Health Belief Model theory: (1) susceptibility of the pregnant women and fetus to malnutrition due to inappropriate dietary practices nutrient deficiency or over nutrient intake; (2) severity of malnutrition such as wasting/thinness and overweight/obesity and high risk of fetus to intrauterine growth retardation, brain development, and cognitive function due to macro- and micronutrient deficiency; (3) benefits of right eating or dietary practices on women nutritional status and fetus health, (4) barriers to practice appropriate good dietary practices; and (5) self-confidence/efficacy to follow right dietary practices. The education was provided using theoretical session, poster, brochures, flipchart, and whiteboard.

For the control group, nutrition education was given by trained community health volunteers based on the general usual nutrition education which is currently provided by health extension workers. Education was given at baseline for three consecutive days. Effect of poor nutrition on maternal and child nutritional status and health was clearly explained. The causes of malnutrition were also briefly explained. Moreover, mothers were clearly informed how they can improve dietary practices, nutritional status, and reduce the burden of malnutrition. The educators encouraged pregnant women to grasp knowledge, provide behavioral change through delivering appropriate nutrition information. After providing nutrition education at baseline, five-month follow-up for was done without providing any additional nutrition education.

### 2.12. Data Processing and Analysis

The questionnaires were coded and entered into SPSS version 20.0. Data were checked for any missing values. Continuous data were checked for normality using the Kolmogrov–Smirnov test. Descriptive statistics such as mean, standard deviation, and percentage were generated. Chi-square test was used to analyze categorized variables. Paired *t*-test for continuous variables and McNemar's test for categorical variables were applied to check significant difference of nutrition knowledge and dietary practice within groups at pre- and postintervention. At 95% confidence level, value with *P* < 0.05 was considered as statistically significant.

## 3. Result

### 3.1. Sociodemographic and Economic Characteristics of Study Participants

Sociodemographic and economic characteristics of the study participants are depicted in Table 1. A total of 138 (69 in each group) pregnant women were participated. About 48 (69.6%) and 46 (66.7%) of participants were within age range of 26–35 years in intervention group (IG) and control group (CG), respectively. About 58 (84.1%) and 61 (88.4) of participants were married and living together with their husbands in IG and CG, respectively. About 18 (26.1%) in IG and 21 (30.4%) of the participants had no formal education. About 39 (56.5%) and 33 (47.8%) of participants had less than four persons per household in IG and CG, respectively. About 14 (20.3%) of respondents' head of household in IG and 17 (24.6%) in CG were not employed. Regarding with monthly income, 15 (21.7%) and 20 (29.0%) of participants had monthly income of less than six hindered Ethiopian birr in IG and CG, respectively. About 35 (50.8%) of participants' cash decision makers were both father and mother in IG while 31 (45.0%) in CG. The chi-square test showed that there was no significance difference (*P* > 0.05) between sociodemographic and economic characteristic of two groups.

The mean and standard deviation (SD) of Health Belief Model (HBM) constructs subscale are shown in Table 2. Except perceiving a barrier, which was decreased, the mean score of all Health Belief Model constructs were increased. The paired *t*-test indicated that there was highly significant difference (*P* < 0.001) between pre- and postnutrition education intervention mean Health Belief Model constructs score in intervention group (IG). The independent *t*-test indicated that there was no significant difference (*P* > 0.05) between mean Health Belief Model construct score of two groups at baseline, but the difference was highly significant (*P* < 0.001) at post nutrition education.

### 3.2. Nutritional Knowledge of Pregnant Women

The mean and standard deviation (SD) score of nutritional knowledge are depicted in Table 3. There was no significant difference (*P*=0.26) between mean nutritional knowledge score of intervention and control groups at baseline, but the difference was highly significant (*P* < 0.001) at the end of nutrition education. The paired *t*-test indicated that the mean nutritional knowledge score was highly significantly improved (*P* < 0.001) from 6.9 to 13.4 in intervention group. Specifically, 100% of participants in intervention group knew sources of macronutrient- and micronutrient-rich foods and consequences of micronutrient deficiency during pregnancy for both mother herself and fetus at the end of nutrition education. At baseline, there was no significant difference (*P* > 0.05) between nutritional knowledge-related variable of two groups, but the difference was significant (*P* < 0.05) at posttest.

### 3.3. Dietary Practices of Pregnant Women

The result of dietary practices is shown in Table 4. The pre- and postintervention good dietary practice was 56.5% and 84.1% in intervention group, and 60.9% and 72.5% in control group, respectively. Specifically, 50.7% of participants in IG and 44.9% in CG experienced food craving at baseline. At the end of intervention, these figures were 28 (40.6) and 26 (37.7%) in experimental and control group, respectively. At baseline, 30 (43.5%) avoided particular foods particularly due to personal dislike (39.1%) and at postintervention 32 (46.4%) avoided due to due to personal dislike (30.4%) intervention in IG. At baseline, about 38 (55.1%) participants in IG ate three main meals and at least one additional snack between meals on daily basis. After nutrition education, 52 (75.4%) of pregnant women ate three meals and at least one additional snack in IG. In control group, 34 (49.3%) and 45 (65.2%) of pregnant women ate three main meals and at least one additional snack between meals on daily basis at pre- and postintervention, respectively.

About 20 (29%) of participants in IG skip at least one main meal at baseline which was decreased to 5 (7.2%) at the end of intervention. On the contrary, 22 (31.9%) and 14 (20.3%) of participants in CG skipped at least one main meal at baseline and endline, respectively. None of the participants ate fresh fruit on daily basis in both groups at baseline. At postintervention, only 2 (2.9%) ate fresh fruits and vegetables on daily basis in IG but none of the participants in CG. About 37 (53.6%) of participants in IG added salt nearly at the end cooking food at baseline which was 67 (97.1%) at postintervention. It was 42 (60.9%) at baseline and 55 (79.7%) at end of the study in CG. At preintervention, about 15 (21.7%) participants followed weight gain in IG and 24 (34.8%) followed weight gain in CG. At postintervention, 53 (76.8%) followed weigh gain in IG and 40 (58.0%) in CG.

The chi-square test showed that there was no significance difference (*P*=0.61) between dietary practice of two groups at preintervention, but the difference was significant (*P*=0.09) at postintervention.

### 3.4. Correlation of Health Belief Model Construct with Dietary Knowledge and Dietary Practices

Except perceived self-efficacy, all other HBM constructs showed highly significant correlation (*P* < 0.001) with dietary practices of pregnant women. Perceived susceptibility, severity, and benefits showed significant positive correlation (*P* < 0.05) with nutritional knowledge (Table 5). Perceived benefits showed negative correlation with both dietary practices and nutritional knowledge. Nutritional knowledge was highly significantly positively correlated (*P* < 0.001) with dietary practice.

## 4. Discussion

This study included 138 pregnant women selected from four clusters. The finding supports the effectiveness of nutrition education based on Health Belief Model (HBM) to improve the nutrition knowledge and dietary practices of pregnant women through increasing perceived severity, susceptibility, benefits, self-efficacy, and decreasing perceived barriers. Accordingly, baseline mean nutritional knowledge score of intervention and control group was 6.9 and 7.4, respectively. At postintervention, the figure was significantly increased to 13.4 in intervention group. The preintervention dietary practice in intervention and control group was 56.5% and 60.9%, respectively. The postintervention result showed that the proportion of pregnant women with good dietary practice increased to 84.1% in intervention group. The improvement in nutritional knowledge and dietary practice within intervention and between two groups was highly statistically significant (*P* < 0.001) at endline.

The finding of present study was in agreement with other studies [27–30] where they suggested that positive effect of nutrition education nutritional knowledge and dietary habits. Another study from southern Ethiopia indicated that providing nutrition education utilizing HBM significantly improved the level of knowledge and attitude of women on household utilization of pulses in intervention group [31]. Another study from Iran reported that providing nutrition education based on HBM significantly improved knowledge, attitude, and practice relating to calcium intake among adolescent students [32]. Similarly, Tavakoli et al. concluded that nutrition education based on HBM has positive significant effect of mean knowledge score, attitude, and behavior in medical student [33]. The finding was also in agreement with another study from Iran in which the authors suggested that providing education based on the Health Belief Model (HBM) can effectively improve the knowledge, attitude, and practice of adolescent students towards nutrient intake. Moreover, educational interventions based on health promotion patterns can be effective in enhancing awareness, better understanding of risks and reducing barriers to healthy behavior, and, ultimately, improving women's health and nutritional performance during pregnancy [34]. Sharifirad et al. also indicated the positive outcome of nutrition education intervention by comparing the effect of nutritional education program based on HBM with traditional education recommended weight gain among pregnant women in Gonabad [35].

The high improvement of nutritional knowledge might be due to frequent education and follow-up during intervention period as well as short interval between the pre- and postassessment. Moreover, the improvement might be explained by increased understanding of the perceived risk and severity of inappropriate nutrition and perceived benefits of practicing good nutrition during pregnancy. On the contrary, the higher improvement of dietary practice intervention group might be explained by higher impact of Health Belief Models by increasing perceived susceptibility, severity, benefits, self-efficacy, and decreased perceived barriers to practice appropriate nutrition and improvement in nutritional knowledge. Women, who fear for severity of malnutrition, understand the benefits of practicing good nutrition for both fetus and herself, and have ability to act on good nutritional activities, were more likely to practice good nutrition. Similarly, women exposed to nutrition information could follow good nutrition practices since research evidence suggested that those women exposed to nutrition information were more likely to practice good dietary practices [36]. Knowledge on severity and susceptibility of mother herself and fetus to malnutrition and poor health outcomes increases; they are likely to be more sensitive to the health of their fetus and infant and follow dietary pattern modifications. On the contrary, improved dietary practice might be explained by improved nutritional knowledge since positive significant correlation was observed with dietary practices.

The slight improvement in nutritional knowledge and dietary practices of control group over time might be due to provision of three-day nutrition education at baseline so that participants practiced as delivered nutrition education information. In addition, currently the health extension workers are delivering basic nutrition counseling while pregnant women attend antenatal care.

This study revealed that except perceived barriers which were decreased, all the other mean Health Belief Model scores significantly increased after nutrition education in experimental group. The perceived barriers were decreased in this study. Apart from perceived barriers, the result of other Health Belief Model constructs' scores was in agreement with finding from Iran [33]. The perceived barriers were increased in compared study. The difference on perceived barriers of two studies might be due to variation in culture, socioeconomic status, and demographic characteristics of two participants. The perceived HBM constructs' scores were in line with other studies [28, 30–33].

Perceived susceptibility and severity showed strong significant correlation (*P* < 0.001), perceived barriers were moderately correlated (*P* < 0.001), and perceived benefits were weakly correlated with dietary practices of pregnant women. This suggested that even if other constructs showed significant correlation, perceived susceptibility and severity have more effect on improving the dietary practices. The negative correlation of perceived barriers with dietary practices indicates that providing nutrition education has a power to improve dietary practices by decreasing barriers for good dietary behaviours. Perceived susceptibility and severity showed moderate significant correlation indicating that they have better effect of improving nutritional knowledge compared to other HBM constructs. The significant correlation of perceived severity and barriers with dietary practices and perceived susceptibility and barriers to nutritional knowledge was in line with a study done Iran [33].

## 5. Conclusion

The overall result indicated that the nutritional knowledge and dietary practices of the pregnant women in intervention group were highly improved compared to their counterparts as well as preintervention results. Hence, delivering nutrition education based on Heath Belief Model can be appropriate tool to improve nutritional knowledge, perceived dietary behaviours, and dietary practice of pregnant women.

## 6. Recommendations

The governmental, nongovernmental organization, health extension workers, and other health-care providers should include Health Belief Model construct into existing nutrition education programs. Moreover, the government should include Health Belief Model constructs in national nutrition education guidelines to bring change in nutritional knowledge and dietary practices of pregnant women.

## Figures and Tables

**Table 1:** Sociodemographic and economic characteristics of the pregnant women in Dessie town, northeast Ethiopia, 2017 GC (*n*_1_=*n*_2_=69).

Variables	Frequency/percentage, *n* (%)		*P* value
	Intervention group	Control group	
Age			
19–25 years	16 (23.1)	21 (30.4)	
26–35 years	48 (69.6)	46 (66.70)	0.11
>35 years	5 (7.2)	2 (2.9)	

Marital status			
Unmarried	11 (15.9)	8 (11.6)	0.29
Married	58 (84.1)	61 (88.4)	

Ethnicity			
Amhara	42 (60.9)	48 (69.6)	0.62
Tigray	13 (18.9)	10 (14.5)	
Afar	7 (10.1)	4 (5.8)	
Others	7 (10.1)	7 (10.1)	

Religion			
Orthodox	41 (59.4)	44 (63.8)	0.98
Muslim	19 (27.5)	18 (26.1)	
Protestant	9 (13.1)	7 (10.1)	

Head of household			
Father	58 (84.1)	61 (88.4)	0.49
Mother	8 (11.6)	4 (5.8)	
Others	3 (4.3)	4 (5.8)	

Educational status of the participants			
No formal education	18 (26.1)	21 (30.4)	0.59
Elementary school attended	14 (20.3)	13 (18.9)	
High school completed	22 (31.9)	19 (27.5)	
Higher institution completed	15 (21.7)	16 (23.2)	

Family income			
<600ETB	15 (21.7)	20 (29.0)	0.69
601–1500ETB	26 (37.7)	23 (33.3)	
>1500ETB	28 (40.6)	26 (37.7)	

Cash decision maker			
Father alone	19 (27.5)	21 (30.4)	0.19
Mother alone	15 (21.7)	17 (24.6)	
Father and mother	35 (50.8)	31 (45.0)	

Family size			
≤4 persons	39 (56.5)	33 (47.8)	0.73
>4 persons	30 (43.5)	66 (52.2)	

Household facilities			
No radio/television	7 (10.1)	10 (14.5)	0.26
Radio/television	62 (89.9)	59 (85.5)	

Occupation of the head of household			
Not employed	14 (20.3)	17 (24.6)	0.84
Government employed	22 (31.9)	21 (30.4)	
Laborer	9 (13.0)	12 (17.4)	
Others	24 (25)	19 (27.5)	

Have source nutrition information			
No	25 (36.2)	28 (40.6)	0.56
Yes	44 (63.8)	41 (59.4)	

Health Belief Model constructs score.

**Table 2:** Health Belief Model constructs score of the pregnant women in Dessie town, northeast Ethiopia, 2017 GC (*n*_1_=*n*_2_=69).

HBM Constructs	Study period	HBM constructs score		*P* value^*∗*^
		Intervention group	Control group	
Perceived susceptibility	Baseline	17.9 ± 4.4	18.5 ± 4.3	0.29
	Endline	28.1 ± 2.1	20.5 ± 5.6	<0.001
	*P* value^b^	<0.001	=0.003	

Perceived severity	Baseline	25.2 ± 6.6	26.7 ± 6.8	0.20
	Endline	37.4 ± 2.6	28.6 ± 7.8	0.001
	*P* value^b^	<0.001	=0.05	

Perceived benefits	Baseline	19.5 ± 4.5	18.2 ± 5.0	0.14
	Endline	23.6 ± 1.4	19.2 ± 5.2	0.001
	*P* value^b^	<0.001	=0.05	

Perceived barriers	Baseline	23.1 ± 4.7	23.1 ± 4.5	0.98
	Endline	16.2 ± 4.2	21.9 ± 5.3	0.001
	*P* value^b^	<0.001	=0.013	

Perceived self-efficacy	Baseline	14.8 ± 3.7	13.7 ± 4.3	0.10
	Endline	17.6 ± 1.9	14.1 ± 4.1	0.001
	*P* value^b^	<0.001	=0.285	

a = independent *t*-test; b = paired *t*-test; HBM = Health Belief Model.

**Table 3:** Nutrition knowledge of the pregnant women in Dessie town, northeast Ethiopia, 2017 GC (*n*_1_=*n*_2_=69).

Knowledge variables	Stud period	Frequency and percentage, *n* (%)		*P* value
		Intervention group	Control group	
Know balanced diet	Baseline	24 (34.8)	27 (39.1)	0.59
	Endline	66 (95.7)	36 (52.2)	<0.001

Know benefits of balanced diet for fetus and herself	Baseline	28 (40.6)	31 (44.9)	0.61
	Endline	62 (89.9)	38 (55.1)	<0.001

Know dietary source of macronutrient (protein, carbohydrate, EFA) rich foods	Baseline	54 (78.3)	56 (81.6)	0.67
	Endline	69 (100)	61 (88.4)	0.004

Know dietary sources of micronutrient(iron, vitamin A, iodine, vitamin C) rich foods	Baseline	22 (31.9)	22 (31.9)	1.00
	Endline	69 (100)	50 (72.5)	<0.001

Know consequences of micronutrient deficiency during pregnancy	Baseline	26 (33.3)	33 (15.9)	0.23
	Endline	65 (100.0)	44 (84.1)	0.007

Know appropriate dietary practice	Baseline	23 (33.3)	32 (46.4)	0.08
	Endline	57 (82.6)	33 (47.8)	<0.001

Know synergetic effect of nutrition and infection	Baseline	38 (55.1)	36 (52.2)	0.73
	Endline	67 (97.1)	45 (65.2)	<0.001

	Mean knowledge score			*P* value^a^

Mean ± SD	Baseline	6.9 ± 2.8	7.4 ± 3.1	0.26
	Endline	13.4 ± 3.1	9.8 ± 3.0	<0.001

*P* value^b^		<0.001	=0.001	

a = independent *t*-test; b = paired *t*-test.

**Table 4:** Dietary practice-related variables among pregnant women in Dessie town, northeast Ethiopia, 2017 GC (*n*_1_=*n*_2_=69).

Variables	Intervention group		Control group	
	Baseline	Endline	Baseline	Endline
Crave food not normally consumed				
Yes	35 (50.7)	28 (40.6)	31 (44.9)	26 (37.7)

Avoided any food items and reasons for avoidance				
Personal dislike	27 (39.1)	21 (30.4)	18 (26.1)	16 (23.2)
Religion	3 (4.3)	6 (8.7)	4 (5.8)	4 (5.8)
Makes fetus big	—	5 (7.2)	2 (2.9)	1 (1.4)

Follow specific dietary regime				
Yes	21 (30.4)	25 (36.2)	29 (42.0)	37 (53.6)

Get 3 meals per day and at least one snacks between main meals				
Yes	38 (55.1)	52 (75.4)	34 (49.3)	45 (65.2)

Skip any of the following meals during current pregnancy				
Dinner	10 (14.5)	1 (1.4)	11 (15.9)	9 (13.0)
Breakfast	9 (13.1)	4 (5.8)	9 (13.0)	4 95.8)
Lunch	1 (1.4)	—	2 (2.9)	1 (1.4)

Eat protein-rich foods on daily basis				
Yes	48 (69.6)	64 (92.8)	52 (75.4)	59 (85.5)

Habits of eating fresh fruits and vegetables on daily basis				
Yes	0 (0.0)	2 (2.9)	0 (0.0)	0 (0.0)

Use iodized salt and add at the end of cooking				
Yes	37 (53.6)	67 (97.1)	42 (60.9)	55 (79.7)

Amount of fluid drunk per day				
Less than 1.5 liter	54 (78.3)	36 (52.2)	50 (72.5)	45 (65.2)
More than 1.5 liter	15 (21.7)	33 (47.8)	19 (27.5)	24 (34.8)

Include milk, fruit juice, soup, and/or other nonalcoholic drinks daily				
Yes	69 (100)	69 (100)	69 (100)	69 (100)

Follow weight during current pregnancy				
Yes	15 (21.7)	53 (76.8)	24 (34.8)	40 (58.0)

Drink alcohol during current pregnancy				
Yes	24 (34.8)	17 (24.6)	24 (34.8)	24 (34.8)

Study period	Group			
	Dietary practice	Intervention	Control	*P* value

Baseline	Good	39 (56.5)	42 (60.9)	0.61
	Poor	30 (43.5)	27 (39.1)	

Endline	Good	58 (84.1)	50 (72.5)	0.09
	Poor	11 (15.9)	19 (27.5)	

**Table 5:** Correlation of Health Belief Model constructs with dietary practice and nutritional knowledge of pregnant women in Dessie town, northeast Ethiopia, 2017 GC (*n*_1_=*n*_2_=69).

Variables	Perceived susceptibility	Perceived severity	Perceived benefits	Perceived barriers	Perceived self-efficacy	Dietary practice	Nutritional knowledge
Perceived susceptibility	1						
Perceived severity	0.900	1					
	*P* < 0.001						
Perceived benefits	0.741	0.657	1				
	*P* < 0.001	*P* < 0.001					
Perceived barriers	−0.795	−0.687	0.770	1			
	*P* < 0.001	*P* < 0.001	*P* < 0.001				
Perceived self-efficacy	0.628	0.628	0.919	−0.601	1		
	*P* < 0.001	*P* < 0.001	*P* < 0.001	*P* < 0.001			
Dietary practice	0.588	0.608	0.342	−0.412	0.253	1	
	*P* < 0.001	*P* < 0.001	*P* < 0.001	*P* < 0.001	*P* = 0.36		
Nutritional knowledge	0.427	0.424	0.214	−0.300	0.149	0.419	1
	*P* < 0.001	*P* < 0.001	*P* = 0.077	*P* = 0.012	*P* = 0.221	*P* < 0.001	

## Data Availability

Datasets used and/or analyzed during the current study are available from the corresponding author on reasonable request.
